# Cu Patterning Using Femtosecond Laser Reductive Sintering of CuO Nanoparticles under Inert Gas Injection

**DOI:** 10.3390/ma14123285

**Published:** 2021-06-14

**Authors:** Mizue Mizoshiri, Kyohei Yoshidomi

**Affiliations:** Department of Mechanical Engineering, Nagaoka University of Technology, Niigata 940-2188, Japan; s193097@stn.nagaokaut.ac.jp

**Keywords:** Cu pattern, CuO nanoparticle ink, femtosecond laser reductive sintering, inert gas injection

## Abstract

In this paper, we report the effect of inert gas injection on Cu patterning generated by femtosecond laser reductive sintering of CuO nanoparticles (NPs). Femtosecond laser reductive sintering for metal patterning has been restricted to metal and metal-oxide composite materials. By irradiating CuO-nanoparticle paste with femtosecond laser pulses under inert gas injection, we intended to reduce the generation of metal oxides in the formed patterns. In an experimental evaluation, the X-ray diffraction peaks corresponding to copper oxides, such as CuO and Cu_2_O, were much smaller under N_2_ and Ar gas injections than under air injection. Increasing the injection rates of both gases increased the reduction degree of the X-ray diffraction peaks of the CuO NPs, but excessively high injection rates (≥100 mL/min) significantly decreased the surface density of the patterns. These results qualitatively agreed with the ratio of sintered/melted area. The femtosecond laser reductive sintering under inert gas injection achieved a vacuum-free direct writing of metal patterns.

## 1. Introduction

Direct laser writing of metals is a promising technique for wiring and fabricating electric devices because it achieves both metallization and formation of the desired patterns. In laser direct writing, various inks based on metal nanoparticles (NPs), metal complexes, metal–organic decompositions, and metal-oxide NPs are coated on substrates, and then selectively metallized by irradiation with focused laser light. Inks that comprised noble metal (such as Au and Ag) NPs are commercially available and achieve highly electroconductive patterns in ambient atmospheres [1,2]. 

Meanwhile, Cu patterning is a potential candidate for a low-cost printing [3]. Presently, Cu nanoparticle inks are selectively sintered by continuous-wave (CW) lasers or by nanosecond, picosecond, and femtosecond pulsed lasers [4,5,6,7]. However, unlike noble metal patterns, Cu patterns are not easily formed in air because the Cu NPs in the inks and fabricated patterns are easily oxidized by the atmospheric oxygen. Therefore, the development of various inks based on Cu-organic decomposition, Cu complexes, and Cu-oxide NPs are carried out. Cu patterns are formed through laser light-induced thermochemical reduction. For example, Cu-organic decomposition and Cu-complex inks coated on substrates are selectively reduced by irradiating them with a CW or pulsed laser, which precipitates the Cu [8,9,10]. In Cu-organic decomposition ink, Cu patterns are formed from Cu(II) formate by a thermal reaction. The gases generated in the decomposition process (CO_2_ and H_2_) provide a reductive atmosphere that prevents the oxidation of the Cu patterns [10]. A glyoxylic acid Cu complex ink for Cu laser direct writing in air has also been developed. On glass substrates coated with this ink, Cu patterns without significant oxidation are formed by a CO_2_ laser-induced thermochemical reaction [8,9]. Other promising candidates for Cu pattering are Cu-oxide NP inks that comprised Cu-oxide NPs, a reductant, and a dispersant. When mixed with a reductant and a dispersant, Cu_2_O NPs are reduced to Cu by a laser-induced thermochemical reaction (where the laser is CW, nanosecond-pulsed, or femtosecond pulsed [11,12,13]). In addition, CuO nanoparticles are also reduced and sintered by various lasers, such as continuous-wave, nanosecond, and femtosecond pulse lasers with green and near-infrared wavelength [3,14,15,16,17,18,19,20]. The irradiated laser light reduces and sinters the Cu-oxide NPs while generating various gases such as CO, CO_2_, and H_2_O. The advantage of the use of Cu-oxide NP inks is their small volume change after irradiation with laser light. The volume remains more-or-less constant because the Cu in the inks has a higher volumetric composition than the Cu precipitated from Cu-organic decomposition and Cu-complex inks. Thermochemical reduction of CuO NPs using CW and nanosecond lasers generates Cu patterns with no significant oxidation [3,14]. The advantage of the femtosecond laser reductive sintering is that the CuO NPs were reductive-sintered under low pulse energy [15,17,21] by comparing to the use of CW and nanosecond pulsed lasers [3,14]. By contrast, reductive sintering with femtosecond lasers selectively form Cu-and Cu_2_O-rich patterns [15,17,21]. In the X-ray diffraction (XRD) spectra of Cu-rich patterns fabricated under femtosecond laser pulses, significant peaks corresponding to Cu_2_O and CuO are found, whereas the Cu patterns obtained by irradiation of CW and nanosecond lasers are almost single-phase Cu. These results typify femtosecond laser reductive sintering of Cu patterning on glass and polyimide film substrates and are commonly reported [15,17,21]. As the oxidation increases with decreasing laser scanning speed [15,17], we considered that Cu becomes re-oxidized by atmospheric oxygen.

In the present study, we investigated the effect of an atmospheric gas injection on reductive sintering of CuO NPs under femtosecond laser pulses. The patterns fabricated under various gases (air, and inert gases of N_2_, and Ar generally used in 3D printing and additive manufacturing) were examined for their crystal structures, surface oxidation, and morphology, and their electric resistances were evaluated.

## 2. Experimental Methods

### 2.1. Cu Patterning Based on Femtosecond Laser Reductive Sintering of CuO NPs under a Controlled Atmosphere

**Figure 1:**
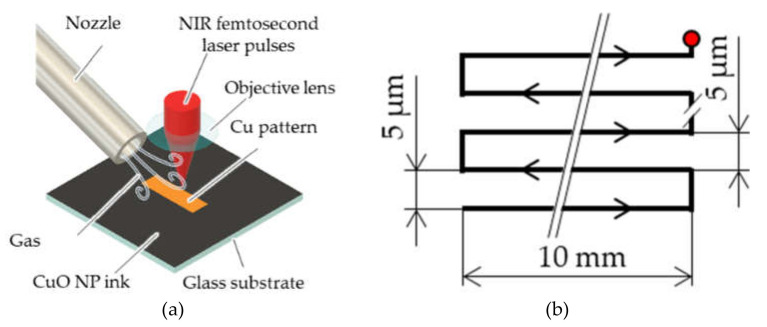
Schematic illustration of Cu patterning under a controlled atmosphere: (**a**) laser patterning and (**b**) scanning route of the focal spot over the paste.

Figure 1 shows the proposed for Cu-patterning process based on femtosecond laser reductive sintering. First, a CuO-NP ink was prepared by using a previously reported process [14,15,17]. CuO NPs (<50 nm diameter, Sigma Aldrich, St. Louis, MS, USA) were dispersed in a mixed solution of polyvinylpyrrolidone (PVP, M_w_ ~10,000, Sigma Aldrich, MS, USA) and ethylene glycol (EG, Sigma Aldrich, MS, USA), and the resulting ink was spin-coated on glass substrates (thickness of ~1 mm). Before spin coating, the substrates were surface-treated with ultraviolet light for 1 min in air to improve their wettability (FLAT EXCIMER EX-mini, Hamamatsu Photonics, Hamamatsu, Japan). The surface of the glass substrates was changed to be hydrophilic by irradiating ultraviolet light which generated ozone from oxygen in air [22]. The CuO-NP ink film coated on the glass was irradiated with near-infrared femtosecond laser pulses with a wavelength, pulse duration, and repetition frequency of 780 nm, 120 fs, and 80 MHz, respectively, (Toptica, FemtoFiber pro NIR, TOPTICA Photonics AG, Munich, Germany). The laser pulses were focused through an objective lens with a numerical aperture of 0.45. The laser irradiation process was conducted under a controlled atmosphere of air, Ar gas, or N_2_ gas injection. The injection rates of the gases were verified using a flow meter. Finally, the non-sintered NPs in the ink were removed by sequentially rinsing the sample in EG and followed by in ethanol. 

### 2.2. Evaluation Methods

The crystal structures of the Cu patterns were examined by XRD analysis using Cu-Kα radiation with a scan speed of 5°/min. The line width was measured by using an optical microscope (Keyence, VE-8800, Osaka, Japan). The morphology of the patterns was also evaluated by using the same optical microscope and a scanning electron microscope (SEM) (HITACHI High Technology Corporation, FlexSEM 1000 II, Tokyo, Japan). The atomic composition and surface oxidation of the patterns were investigated using an energy-dispersive X-ray spectroscopy (EDS, Oxford Instruments plc, Abingdon-on-Thames, United Kingdom). Additionally, a laser Raman spectrometer (JASCO, NRS-7200, Tokyo, Japan) with a laser wavelength of 532 nm, respectively. The resistances of the patterns were measured using a multimeter (Keysight Technology, Truevolt series 34465A, Santa Rosa, CA, USA).

## 3. Results

### 3.1. Effect of Air Injection on the Pattern Uniformity 

**Figure 2:**
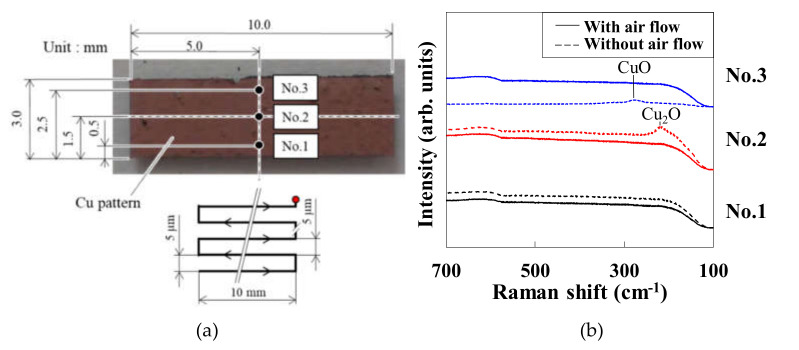
(**a**) Photograph of the pattern fabricated under an air injection at a scan speed and pulse energy of 5 mm/s and 0.74 nJ, respectively; (**b**) Raman spectra at points No. 1, No. 2, and No. 3 of the patterns fabricated in the presence and absence of air injection.

We first investigated the effect of gas injection on the pattern uniformity. Figure 2a is a photograph of the patterns fabricated with and without air injection using compressed air with controlling the flow rate, and Figure 2b shows the Raman spectra of the patterns. The patterns were fabricated at a scan speed and pulse energy of 5 mm/s and 0.74 nJ, respectively. The Raman spectroscopy was performed at measurement points No. 1, No. 2, and No. 3, in Figure 2a. In the patterns written at the starting area (No. 1), the CuO NPs were highly reduced and significant oxidation-free regardless of air injection (absent or present). By contrast, at points No.2 and No.3, copper-oxidized Cu_2_O and Cu species, respectively, were found in the absence of air injection, but significant oxidation was not observed under air injection. These results suggest that air injection improves the uniformity of the beginning and end of the written area. The thermochemical reduction of CuO NPs to Cu creates water vapor that coats the surface of the objective lens, decreasing the laser pulse energy irradiated on the CuO NP paste and hence the degree of the reduction to Cu [14].

### 3.2. Effect of Scan Speed on the Crystal Structures of the Patterns

**Figure 3:**
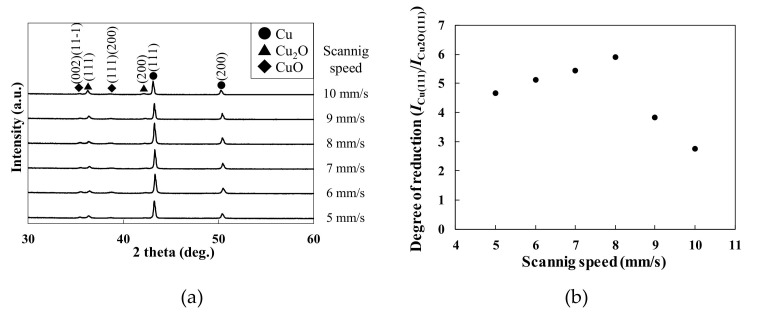
(**a**) XRD spectra of the patterns fabricated at various scan speeds under an air injection; (**b**) degree of reduction at various scan speeds.

We next investigated the effect of scan speed on the crystal structures. Figure 3a shows the XRD spectra of the patterns fabricated at different scan speeds. Intense peaks corresponding to Cu were observed in all spectra. Small peaks corresponding to Cu_2_O and CuO were also observed. Figure 3b plots the degree of reduction as the intensity ratio of Cu(111)/CuO(111) versus the scan speed. The degree of reduction was maximized at 8 mm/s and was relatively small at higher and lower scan speeds. These results suggest low reduction and the re-oxidation of the patterns at high and low scan speeds, respectively.

### 3.3. Crystal Structures of the Patterns Fabricated under Various Gas Injections

**Figure 4:**
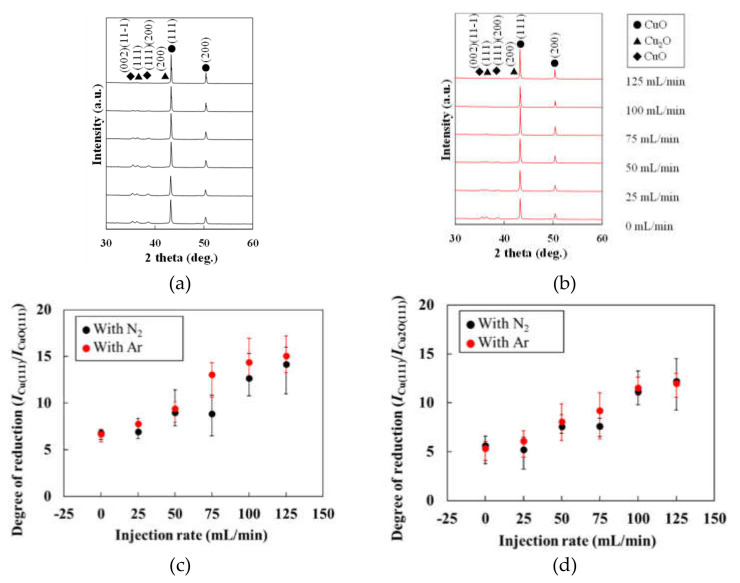
XRD spectra of the patterns fabricated under (**a**) N_2_ gas and (**b**) Ar gas; degrees of reduction, (**c**) Cu/CuO and (**d**) Cu/Cu_2_O of the patterns fabricated under gases with various injection rates.

This subsection investigates the crystal structures of the patterns fabricated under N_2_ and Ar gas injections. Figure 4a,b show the XRD spectra of the patterns fabricated under N_2_ and Ar gas injections, respectively. The XRD peaks corresponding to CuO and Cu_2_O decreased in both XRD spectra of both patterns. Figure 4c,d plot the degree of reductions, reported as the XRD intensity ratio *I*_Cu(111)_/*I*_CuO(111)_ and *I*_Cu(111)_/*I*_Cu2O(111)_ of the patterns formed under various gas injection rates. The Cu generation in the patterns increased with gas injection rate. The degree of the reduction was not affected by the types of gasses. This result suggests that both gases removed O_2_ gas from atmospheric air during sintering and melting of the CuO NPs. 

### 3.4. Surface Morphology

**Figure 5:**
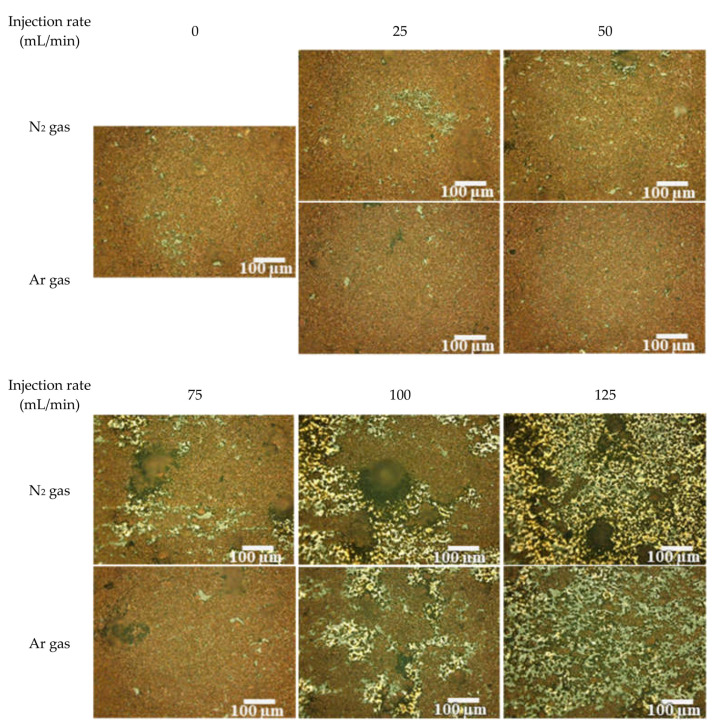
Optical microscope images of the patterns fabricated under various N_2_ and Ar gases at different injection rates.

**Figure 6:**
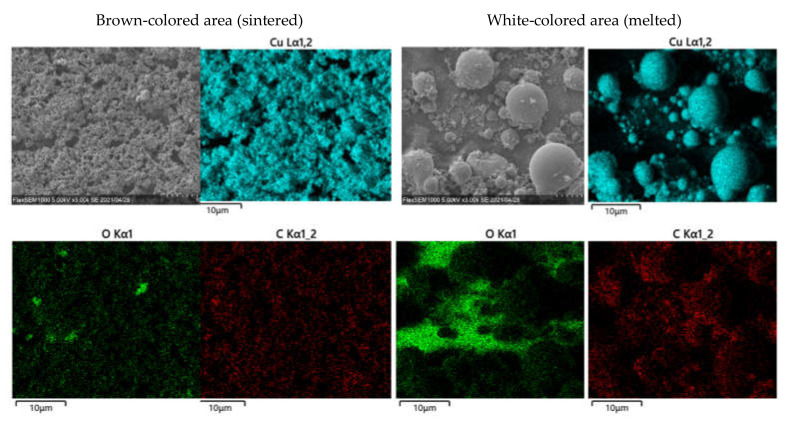
SEM images and EDS mappings of patterns fabricated under Ar gas injection rates of 125 mL/min.

The surface morphologies of the patterns fabricated under N_2_ and Ar gases at various injection rates are revealed in the optical images (Figure 5). The copper-like brown and pore-like white area were observed under the injection rate of ≥100 mL/min. Figure 6 shows the SEM image EDS mappings of the brown-colored and white-colored areas of the pattern fabricated under the Ar gas injection rate of 125 mL/min, revealing that the brown-colored area was well-sintered and white-colored area was the bare glass substrates because the white-colored area was not Cu- and O-rich, established through EDS mapping, corresponding to glasses. The droplets were generated in the melted area. Table 1 lists the surface densities of the fabricated patterns. The Cu metal luster of the patterns were observed in all the optical images. The density decreased with the increasing injection rate of both gases. The patterns formed under high injection rates (100 and 125 mL/min) exhibited many droplets that apparently formed by the melting and cooling of the materials. These droplets were considered to markedly decrease the density of the patterns from those formed at lower injection rates (0–75 mL/min).

**Figure 7:**
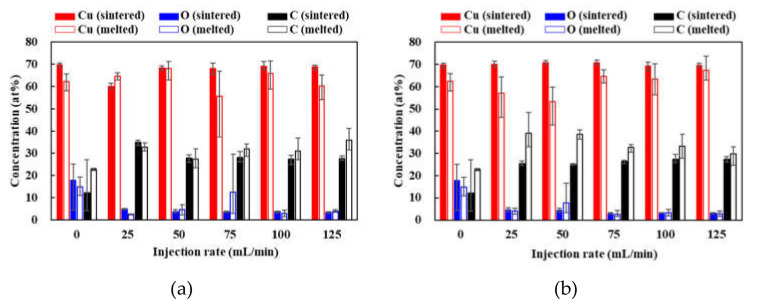
Concentration ratio of the patterns in sintered and melted area under (**a**) N_2_ and (**b**) Ar gas injections.

Figure 7 shows the concentration ratio of the patterns in sintered and melted area. The concentration of Cu in the sintered area was approximately 70% which was not affected by the kinds of gases and their injection rate. The concentration of oxygen decreased with increasing the injection rate, that of carbon increased with an increase in both the sintered and melted area. These results agreed with the degree of reductions, Cu/CuO and Cu/Cu_2_O shown in Figure 4.

### 3.5. Resistance

**Figure 8:**
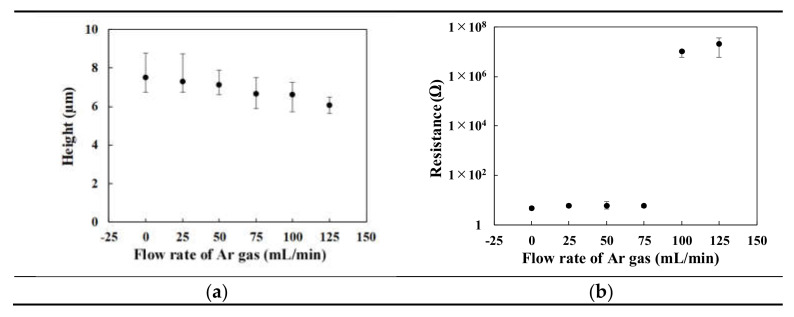
(**a**) Height and (**b**) resistance of the patterns fabricated under various injection rate of Ar gas.

The resistances of the patterns were determined along their cross-sections. Figure 8a shows the height of the patterns fabricated at various injection rate of Ar gas. The resistance of the patterns fabricated under various gas injection rates is shown in Figure 8b. The resistance significantly increased over 100 mL/min of the Ar gas. This result qualitatively agreed with the ratio of sintered/melted area of the patterns as shown in Table 1. By improving the density by optimizing a concentration of CuO NPs or a size distribution of the CuO NPs in the CuO NP paste, the higher electrical conductivity of the patterns is expected to be formed by femtosecond laser reductive sintering of CuO NPs.

## 4. Discussion

The concentration of oxygen decreased with increasing the gas injection rate by XRD and EDS analyses. In contrast, the resistance of the patterns fabricated under high gas injection rate of ≥100 mL/min, increased with increasing the gas injection rate. By considering the porosity of the patterns, the high resistance of the patterns was induced by the low density of the patterns. These results indicate that the purity of the patterns was improved by increasing the gas injection rate.

The advantage of the femtosecond laser reductive sintering is that the CuO NPs were reductive-sintered under low pulse energy of ~0.5 nJ, namely low average power of ~50 mW, by comparing to the use of continuous-wave and nanosecond pulsed lasers of ~0.2 W [14]. However, the concentration of the oxide in the patterns fabricated using femtosecond laser pulses [15,17,21] was higher than the patterns fabricated using continuous-wave and nanosecond pulsed lasers by examining the fabricated patterns [14]. We experimentally demonstrated that the high concentration of oxygen in the patterns was caused by the atmospheric oxygen by controlling the ambient atmosphere in the femtosecond laser irradiation process. This result is believed to be important to obtain Cu patterns with high purity and to consider the reduction process of CuO nanoparticles. In the femtosecond laser irradiation process, a large intensity of the femtosecond laser pulses is advantageous to heat the CuO nanoparticles rapidly. In contrast, the rapid heating and cooling processes generated the non-equilibrium material of Cu_2_O in air [15,17,21]. The control of the ambient atmosphere is effective to improve the purity of the Cu in the patterns.

## 5. Conclusions

We evaluated the effect of the inert gas injections on the patterns fabricated by femtosecond laser reductive sintering of CuO nanoparticles.

(1) The XRD and EDS analysis suggest that the purity of the Cu in the patterns was improved by increasing the gas injection rate.

(2) The resistance of the patterns fabricated under excessively high injection rates (≥100 mL/min) increased. By considering the density of the patterns, the high resistance was caused by the low density of the patterns and not high oxidation.

These results suggest that the rapid heating and cooling in the femtosecond laser reductive sintering of CuO NPs generated the non-equilibrium materials of Cu_2_O due to the reoxidation in air, which was different from the irradiation of continuous-wave and nanosecond pulsed lasers to the CuO NP inks. The concentration of the oxygen in the patterns can be reduced by irradiating the femtosecond laser pulses under inert gas injection.

## Figures and Tables

**Table 1:** The ratio of sintered/melted area of the patterns fabricated under N_2_ and Ar gases at different injection rates.

Injection Rate (mL/min)	0	25	50	75	100	125
N_2_ gas	95.1%	94.5%	94.0%	93.2%	81.8%	68.8%
Ar gas	95.9%	96.4%	96.6%	95.7%	84.9%	76.9%
